# Low Density Lipoprotein Receptor-Related Protein-1 (LRP1) Is Involved in the Uptake of *Clostridioides difficile* Toxin A and Serves as an Internalizing Receptor

**DOI:** 10.3389/fcimb.2020.565465

**Published:** 2020-10-19

**Authors:** Dennis Schöttelndreier, Anna Langejürgen, Robert Lindner, Harald Genth

**Affiliations:** ^1^Institutes for Toxicology, Hannover Medical School, Hannover, Germany; ^2^Neuroanatomy and Cell Biology, Hannover Medical School, Hannover, Germany

**Keywords:** clostridial glycosylating toxins, endocytosis, receptors, cell surface, clostridioides difﬁcile infection

## Abstract

Toxin producing *Clostridioides difficile* strains cause gastrointestinal infections with the large glucosylating protein toxins A (TcdA) and B (TcdB) being major virulence factors responsible for the onset of symptoms. TcdA and TcdB enter their target cells *via* receptor-mediated endocytosis. Inside the cell, the toxins glucosylate and thereby inactivate small GTPases of the Rho-/Ras subfamilies resulting in actin reorganization and cell death. The receptors of TcdA are still elusive, glycoprotein 96 (gp96), the low density lipoprotein receptor family (LDLR) and sulfated glycosaminoglycans (sGAGs) have most recently been suggested as receptors for TcdA. In this study, we provide evidence on rapid endocytosis of Low density lipoprotein Receptor-related Protein-1 (LRP1) into fibroblasts and Caco-2 cells by exploiting biotinylation of cell surface proteins. In contrast, gp96 was not endocytosed either in the presence or absence of TcdA. The kinetics of internalization of TfR and LRP1 were comparable in the presence and the absence of TcdA, excluding that TcdA facilitates its internalization by triggering internalization of its receptors. Exploiting fibroblasts with a genetic deletion of LRP1, TcdA was about one order of magnitude less potent in LRP1-deficient cells as compared to the corresponding control cells. In contrast, TcdB exhibited a comparable potency in LRP1-proficient and -deficient fibroblasts. These findings suggested a role of LRP1 in the cellular uptake of TcdA but not of TcdB. Correspondingly, binding of TcdA to the cell surface of LRP1-deficient fibroblasts was reduced as compared with LRP1-proficient fibroblasts. Finally, TcdA bound to LRP1 ligand binding type repeat cluster II (amino acid 786–1,165) and cluster IV (amino acid 3332-3779). In conclusion, LRP1 appears to serve as an endocytic receptor and gp96 as a non-endocytic receptor for TcdA.

## Introduction

*Clostridioides difficile*, the leading cause of pseudomembranous colitis and hospital acquired diarrhea, produces two exotoxins, toxin A (TcdA) and toxin B (TcdB), as its major virulence factors (Aktories et al., 2017; Chandrasekaran and Lacy, 2017). TcdA and TcdB are single-chained protein toxins that enter their mammalian target cells by receptor-mediated endocytosis. Cell entry is initiated by toxin binding to the cell surface (mediated by the C-terminal binding domains), followed by internalization in the endosome, and translocation of the N-terminal glucosyltransferase domain (GTD) through a pore in the endosomal membrane formed by an intermediate pore forming domain. Once in the cytosol, the GTD mono-O-glucosylates and thereby inactivates small GTPases of the Rho/Ras subfamilies (Genth et al., 2018). In cultured cells, toxin-catalyzed inactivation of Rho/Ras GTPases results in actin de-polymerization, cell cycle arrest, and cell death (May et al., 2013; Beer et al., 2018).

Although TcdA and TcdB exhibit a comparable domain structure and both enter the cell by receptor-mediated endocytosis, their pathways of cellular uptake are different (Papatheodorou et al., 2018). Both toxins exploit different receptors for cell surface binding and internalization, based on different binding properties to their receptors and different sensitivity among cell types (Papatheodorou et al., 2010; Chandrasekaran et al., 2016). For TcdB, three protein receptors have been proposed including Chondroitin sulfate proteoglycan 4 (CSPG4), Poliovirus receptor-like 3 (PVRL3) and Frizzled 1/2/7 (LaFrance et al., 2015; Tao et al., 2016; Gupta et al., 2017; Tao et al., 2019). However, these receptors seems to mediate cell surface binding of TcdB (rather than TcdB internalization), as none of them is internalized in cells to a detectable extent (Schottelndreier et al., 2018). For TcdA, several protein receptor candidates have been suggested, including rabbit sucrose-isomaltase, glycoprotein 96 (gp96), and members of the low density lipoprotein receptor (LDLR) family. Furthermore, the glycan structures Gal-α-1,3-Gal-β-1,4-GlcNAc, Lewis X/Y/I glycans, and sulfated glycosaminoglycans have been suggested as carbohydrate receptors for TcdA (Clark et al., 1987; Tucker and Wilkins, 1991; Pothoulakis et al., 1996; Na et al., 2008; Tao et al., 2019).

The LDLR family includes Low density lipoprotein receptor-related protein-1 (LRP1), also known as CD91 or α-2-macro globulin receptor. LRP1 (widely expressed in multiple cell types) is an endocytic and a signaling receptor that is involved in lipoprotein uptake and lipid metabolism, in atherosclerosis, and in the control of glucose homeostasis (Au et al., 2017). During its maturation, the 600 kDa protein is cleaved by furin in the Golgi complex leaving a 85 kDa C-terminal membrane bound fragment (the light β chain) that is non-covalently attached to extracellular located 515 kDa N-terminal fragment (the heavy α chain). The α chain consists of ligand-binding-type repeats forming 4 clusters that are separated by epidermal growth factor repeats and allow interaction with diverse molecules. LRP1 ligands (including apolipoprotein E, Lipoprotein lipase, coagulation factor VIII light chain, Lactoferrin, plasminogen activator inhibitor (PAI), and tissue plasminogen activator (tPA)) bind to clusters II and IV (Lillis et al., 2008; Herz et al., 2009; Bres and Faissner, 2019).

Glycoprotein 96 (gp96), a member of the hsp90 family of chaperons, is widely expressed in the ER but is also present on the cell surface (Ansa-Addo et al., 2016). Gp96 is involved in folding of multiple secretory and membrane receptor proteins such as integrins, Toll-like receptors and LRP6 (Wu et al., 2012). Additionally, cell surface gp96 is involved in the cell entry for multiple intracellular bacteria like adherent-invasive *E. coli* and *Listeria monocytogenes* (Cabanes et al., 2005; Mittal and Prasadarao, 2011; Rolhion et al., 2011).

The hypothesis behind this study is that LRP1 and gp96 are involved in the cellular uptake of TcdA. This hypothesis is substantiated by recent findings on the involvement of LDLR family members in TcdA uptake (Tao et al., 2019). Furthermore, LRP1 has earlier been proposed as a receptor for several bacterial toxins including the related glucosylating large cytotoxin from *Clostridium perfringens* (TpeL) (Schorch et al., 2014), the vacuolating cytotoxin (VacA) from Helicobacter pylori (Yahiro et al., 2012), and the Pseudomonas Exotoxin A (Pastrana et al., 2005). Exploiting biotinylation of cell surface proteins, we here show that LRP1 is internalized in mouse embryonic fibroblasts (MEFs). TcdA (not TcdB) binds LRP1 cluster IV in a cell-free system. Genetic deletion of LRP1 resulted in reduced cellular uptake and reduced cell surface binding of specifically TcdA. In contrast, neither cellular uptake nor cell surface binding of the related full-length TcdB was affected upon LRP1 deletion.

## Materials und Methods

### Materials

The following reagents were obtained from commercial sources: Sulfo-NHS-SS-Biotin and Neutravidin-agarose were bought from ThermoFisher; glutathione, phenylmethanesulfonyl fluoride (PMSF), E64 and iodacetamide were bought from Sigma; Leupeptin and Pepstatin were bought from Biomol; LRP1 cluster II and cluster IV were bought from R&D Systems.

TcdA and TcdB from *C. difficile* VPI10463 were produced and purified as previously described (Genth et al., 2000). In brief, a dialysis bag containing 900 mL of 0.9% NaCl in a total volume of 4 liters of brain heart infusion (Difco, BD Life Sciences, Heidelberg, Germany) was inoculated with 100 mL of an overnight culture of *C. difﬁcile*. The culture was grown under microaerophilic conditions at 37°C for 72 h. Bacteria were removed from the dialysis bag solution by centrifugation. Proteins from the culture supernatant were precipitated by ammonium sulfate (Merck Millipore, Darmstadt, Germany) at 70% saturation. The precipitated proteins were dissolved in 50 mM Tris-HCl pH 7.5 buffer and extensively dialyzed against 50 mM Tris-HCl pH 7.5 buffer for 24 h. The protein solution was loaded onto an anion exchange column (MonoQ, GE Healthcare Europe, Freiburg, Germany). TcdB was eluted with 50 mM Tris-HCl, pH 7.5, at 500–600 mM NaCl and was subsequently dialyzed against buffer (50 mM Tris-HCl pH 7.5, 15 mM NaCl). The absence of TcdA (which eluted at 150–200 mM NaCl) in TcdB preparations was checked by immunoblot analysis.

### Cell Culture

Murine embryonic fibroblasts (MEF) from C57BL/6 LRP1^+/+^ and LRP1^−/−^ littermate embryos were kindly provided by Joachim Herz (Dallas, Texas, USA) (Zurhove et al., 2008; Schorch et al., 2014). LRP1^+/+^ MEFs and LRP1^-/-^ MEFs were cultured in Dulbecco´s modified essential medium supplemented with 10% FCS, 100 µg/ml penicillin, 100 U/ml streptomycin and 1 mM sodium pyruvate at 37°C and 5% CO_2_. Cells were seeded sub-confluently in 3.5** cm** dishes and treated with the toxins or buffer with indicated concentrations and times as noted in the figures. Upon incubation time, cells were rinsed with 1** ml** ice cold phosphate-buffered saline and scraped off with 200 µl Laemmli lysis buffer per dish. The lysates were subjected to immunoblotting.

### Immunoblotting

Proteins from cell lysates were separated using 15% polyacrylamide gels und transferred onto nitrocellulose for 2 h at 120 V, followed by blocking with 5% (*w/v*) nonfat dried milk for 1 h. Primary antibodies were incubated over night at 4°C with dilution according to the manufacturers´ instructions ((beta-actin, Mab AC-40, Sigma-Aldrich, St. Louis, MO, USA; dilution 1:5,000); PAK2 (Cell signaling 2608, dilution 1:1,000); phospho-S144/141-PAK1/2 (Abcam ab40795; dilution 1:1,000); Rac1 (BD Transduction Laboratories 610650, clone 102; dilution 1:1,000); Rac1 (Millipore 05-389, clone 23A8; dilution 1:1,000); LRP1 (Abcam ab92544; dilution 1:50,000); TfR (Invitrogen 13-6,800, clone H68.4; dilution 1:1,000); gp96 (R&D Systems 816803, dilution 1:1,000) in TBST buffer (50 mM Tris-HCL, pH 7.2, 150 mM NaCl, 5 mM KCl, 0.05% (*w/v*) Tween 20) and subsequently for 1 h at room temperature with HRP-conjugated secondary antibody (mouse: Rockland 610-1034-121; dilution 1:5,000; rabbit Rockland 611-1302; dilution 1:5,000). For the chemiluminescence reaction, ECL Femto (Fisher Scientific, Schwerte, Germany) was used. The signals were detected with the INTAS Chemo Cam Imager (Intas Science Imaging Instruments GmbH, Göttingen, Germany) and analyzed densitometrically using the LabImage 1D software (Kapelan Bio-Imaging GmbH, Leipzig, Germany).

### Cell-Surface Toxin Binding

LRP1^+/+^ MEF and LRP1^-/-^ MEF were seeded subconfluently in 3.5 cm dishes. The cells were chilled to 4°C prior to toxin treatment with either 6 nM TcdA or TcdB for 1 h. Cells were carefully rinsed twice with 1 ml ice cold phosphate-buffered saline and scraped off with 150 µl Laemmli lysis buffer per dish. The lysates were subjected to immunoblot analysis. Toxins were detected with polyclonal anti-toxin IgGs raised in our lab.

### Overlay Assay

For the overlay assay, 5 pmol LRP1 cluster II and cluster IV were immobilized on nitrocellulose membrane and incubated with 3 nM Toxin solution after blocking for 1 h with 5% (*w/v*) nonfat dried milk. Primary antibodies were incubated at room temperature for 2 h followed by HRP-conjugated secondary antibody incubation for 1 h at room temperature before signal detection using the INTAS Chemo Cam Imager (Intas Science Imaging Instruments GmbH, Göttingen, Germany) and analyzed densitometrically using the LabImage 1D software (Kapelan Bio-Imaging GmbH, Leipzig, Germany).

### Cell Surface Biotinylation Internalization Assay

Cell surface biotinylation of MEFs and internalization was performed as described (Knorr et al., 2009). Brieﬂy, cells were detached with 7.5 mM EDTA in PBS for 10–15 min at 37°C, washed and resuspended in Hanks’ balanced salt solution (HBSS). 0.5 mg/ml biotinylation reagent Sulfo-NHS-SS-Biotin (Pierce) was added for 30 min on ice before stopping the reaction with cold culture medium (DMEM) supplemented with 50 mM glycine for 60–75 min. After washing with cold HBSS, endocytosis was started by incubating the labeled cells with 37°C warm endocytosis medium (HBSS supplemented with 1% BSA and 1% FCS (EM)) for 2–16 min at 37°C. Endocytosis was stopped by adding ice-cold EM followed by glutathione-stripping of remaining surface biotin with GSH-stripping buffer (50 mM GSH, 100 mM NaCl, 10% FCS at pH 8,5). Cells were lysed in lysis buffer (50 mM TRIS pH 7.5, 100 mM NaCl, 1% TX-100 supplemented with leupeptin, PMSF, E-64, pepstatin, and iodoacetamide) over night at 4°C. After ultracentrifugation for 45 min at 25,000g, internalized biotinylated proteins were isolated from the supernatant with neutravidin-agarose (ThermoFisher) and subjected to immunoblotting.

## Results

### Internalization of LRP1

**Figure 1:**
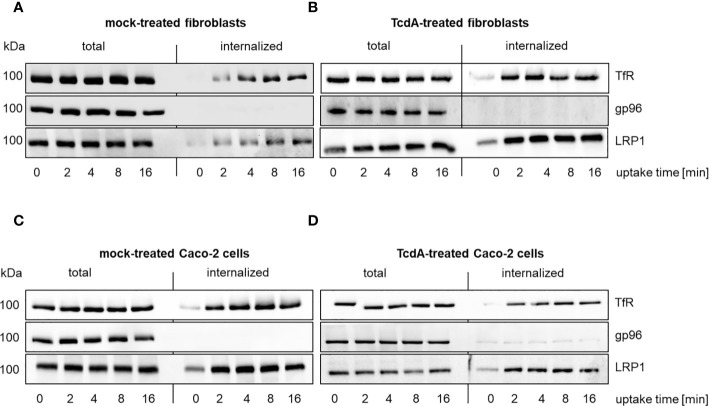
Internalization of LRP1 in murine fibroblasts and human colocytes Caco-2 cells. Internalization of reversibly biotinylated cell surface proteins [transferrin receptor (TfR); glycoprotein 96 (gp96); LDL related protein 1 (LRP1)] into murine fibroblasts **(A, B)** or Caco-2 cells **(C, D)** was induced by temperature shift to 37°C. Cells were either left untreated (total) or exposed to glutathione to remove cell surface biotin (internalized). Cells were exposed to either buffer **(A, C)** or 333 pM TcdA **(B, D)** for 30 min at 37°C and biotinylated proteins were retrieved using neutravidin agarose and analyzed by immunoblotting. Representative immunoblots are shown.

Receptor-mediated endocytosis allows cellular uptake of cargo ligands. To check if LRP1 undergoes endocytosis, LRP1 internalization was analyzed using cell surface biotinylation on ice (Knorr et al., 2009; Schottelndreier et al., 2018). Temperature was shifted to 37°C to allow internalization followed by removal of residual cell surface biotin with glutathione. Transferrin (Tf) binds to the Tf receptor (TfR) and enters the cell through receptor-mediated endocytosis. Inside the cell, Tf is trafficked to early endosomes, where it delivers iron. Internalized TfR and total TfR (cell surface and internalized) were analyzed using immunoblotting. At point in time 0, the signal represents exclusively surface exposed TfR (**Figure 1A**). A part of total TfR rapidly internalized into SV40-immortalized mouse embryonic fibroblasts (MEF) within a few minutes (**Figure 1A**). Rapid internalization of TfR served as a positive control (Knorr et al., 2009).

Comparable to TfR, a part of total LRP1 rapidly internalized into fibroblasts as well (**Figure 1A**, **Figure S1**). Besides LRP1, gp96 has also been suggested as TcdA receptor (Na et al., 2008). In contrast to LRP1, almost no internalization of gp96 was observed (**Figure 1A**). TcdA might act as a ligand (that enhances endocytosis) or TcdA-induced actin de-polymerization might facilitate internalization of gp96. The kinetics of internalization of TfR, gp96, and LRP1, however, were comparable in the presence and the absence of TcdA (**Figure 1B**). The presence of TcdA did thus not trigger internalization of gp96.

Human colonic epithelial (Caco-2) cells represent an often exploited cell culture model in the enterotoxin field. A part of total LRP1 and TfR internalized into mock-treated Caco-2 cells (**Figure 1C**). Comparable to the observations from fibroblasts, no internalization of gp96 was observed in mock-treated and TcdA-treated Caco-2 cells (**Figures 1C, D**).These findings are likely to exclude that TcdA facilitates its internalization by triggering internalization of its receptors. This findings show that LRP1 is an endocytic receptor while gp96 is a non-endocytic receptor.

### Prevention of TcdA Induced Cell Rounding Upon Genetic LRP1 Inhibition

**Figure 2:**
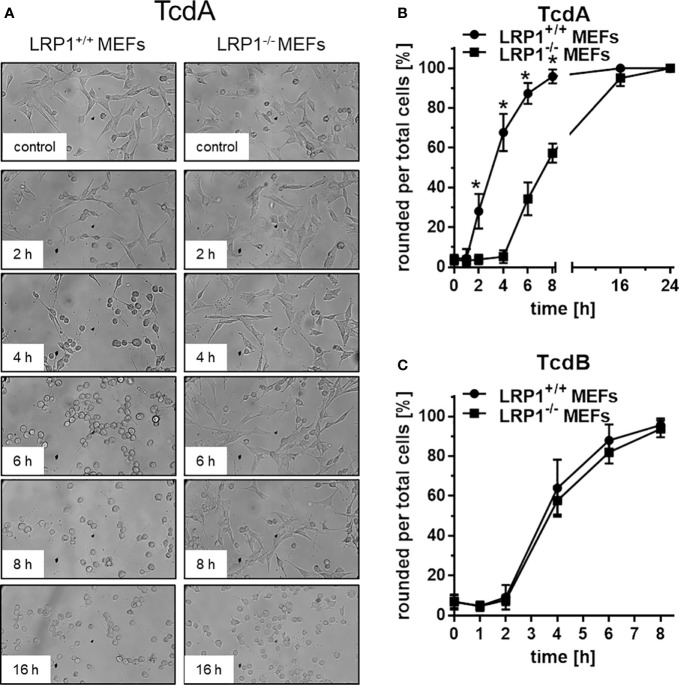
Delayed TcdA-induced actin de-polymerization upon genetic deletion of LRP1. LRP1^-/-^ mouse embryonic fibroblasts (MEFs) and LRP1^+/+^ MEFs were treated with 100 pM TcdA **(A, B)** and 3,7 pM TcdB **(C)** for the indicated times and the cell morphology was visualized using phase contrast microscopy. Toxin-induced actin depolymerization was quantified in terms of the number of rounded per total cells. Values represent the mean ± SD from three independent experiments. * indicates significant differences, p < 0,05 as analyzed using student´s t-test.

TcdA and TcdB induced actin de-polymerization (May et al., 2013), resulting in cell rounding (**Figure 2A**). TcdA and TcdB time-dependently induced rounding of LRP1^+/+^ MEFs (**Figure 2A**), with TcdA or TcdB treatment for 8 h being sufficient for almost complete cell rounding (**Figures 2A–C**). In contrast, TcdA-induced rounding of LRP1^-/-^ MEFs was clearly delayed, albeit complete cell rounding was archieved upon prolonged TcdA treatment for 24h (**Figure 2B**). TcdA was about one order of magnitude less potent in LRP1^-/-^ MEFs as compared to the corresponding LRP1^+/+^ MEFs (**Figure S2A**) whereas TcdB was potent in both LRP1^-/-^ MEFs and LRP1^+/+^ MEFs (**Figure 2C**, **S2B**).

**Figure 3:**
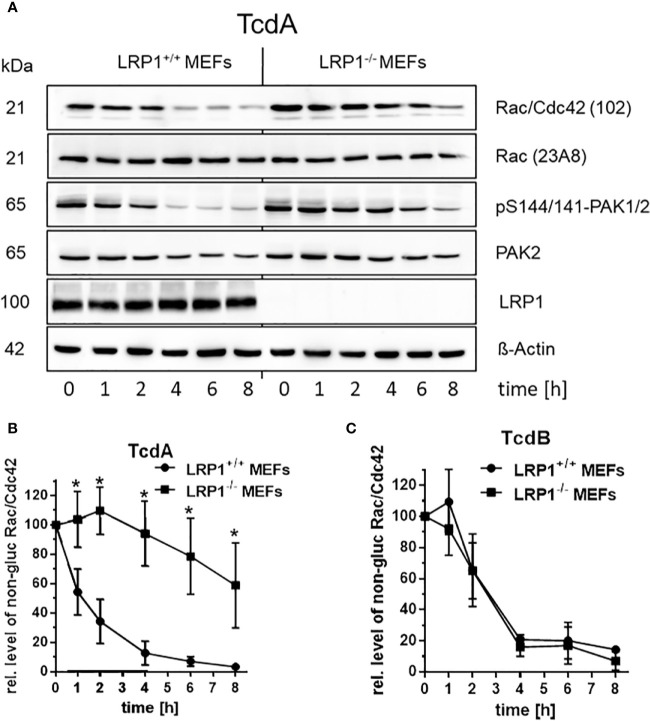
Delayed cellular uptake of TcdA upon genetic deletion of LRP1. **(A)** LRP1^-/-^ MEFs and LRP1^+/+^ mouse embryonic fibroblasts (MEFs) were treated with 100 pM TcdA for the indicated times. The cellular levels of non-glucosylated Rac/Cdc42, total Rac1, pS144/141-PAK1/2, LRP1, and beta actin were analyzed by immunoblotting using the indicated antibodies. **(B)** Quantification of the relative level of non-glucosylated Rac/Cdc42 versus total Rac1 of time-dependent 100 pM TcdA treated MEFs **(C)** and time-dependent 3,7 pM TcdB treated MEFs are expressed as the mean ± SD from three independent experiments. * indicates significant differences, p < 0,05 as analyzed using student´s t-test.

Toxin induced actin de-polymerization results from toxin catalyzed GTPase glucosylation, which is a well-established surrogate marker for toxin uptake (Chandrasekaran et al., 2016; Papatheodorou et al., 2019). Above findings (**Figure 2**) show that deletion of LRP1 reduces the cellular effect of TcdA (not of TcdB), suggesting that cellular uptake of TcdA was delayed. For the analysis of Rac/Cdc42 glucosylation, cell lysates were analyzed by immunoblot analysis using the anti-Rac1 (clone 102) antibody (Genth et al., 2006; Brandes et al., 2012). This antibody is specific for non-glucosylated Rac/Cdc42 subtype GTPases. Once Rac/Cdc42 are glucosylated, the Rac1 (clone 102) antibody does not detect its epitope, resulting in a decreased signal (**Figure 3A**). TcdA time-dependently induced Rac/Cdc42 glucosylation in LRP1^+/+^ MEFs (**Figure 3B**). In TcdA-treated LRP1^-/-^ MEFs, Rac/Cdc42 glucosylatlon was clearly delayed (**Figure 3A**). In the concentration-dependent experiment, TcdA was about factor 10 less potent in LRP1^-/-^ MEFs as compared to LRP1^+/+^ MEFs (**Figure S3A**). In contrast, no significant difference in the kinetics of TcdB-induced Rac/Cdc42 glucosylation was observed between LRP1^-/-^ MEFs and LRP1^+/+^ MEFs (**Figure 3C**, **Figure S3B**). The cellular level of Rac1 was unchanged upon toxin treatment (as analyzed using the Rac1 (clone 23A8) antibody) (**Figure 3A**), confirming that decreasing detection of Rac/Cdc42 subtype GTPases by the Rac1 (Mab 102) antibody was due to glucosylation but not due to degradation.

The Rac/Cdc42 effector kinase p21-associated kinase1/2 (PAK1/2) is a component of focal adhesions and the centrosome (May et al., 2014). Glucosylation of Rac/Cdc42 results in decreased levels of pS144/141-PAK1/2, indicating PAK deactivation (May et al., 2014). TcdA-catalyzed Rac/Cdc42 glucosylation was reflected by decreased levels of pS144/141-PAK1/2 (**Figure 3A**). In TcdA-treated LRP1^-/-^ MEFs, PAK dephosphorylation was clearly delayed (**Figure 3A**). Toxin-induced cell rounding thus nicely correspond to Rac/Cdc42 glucosylation and PAK dephosphorylation. Exploiting Rac/Cdc42 glucosylation and PAK de-phosphorylation as surrogate markers of toxin uptake, genetic deletion of LRP1 resulted in reduced cellular uptake of specifically TcdA, strongly suggesting a role of LRP1 in TcdA uptake. The observation that TcdB was taken up into in LRP1^-/-^ MEFs and LRP1^+/+^ MEFs with similar kinetics was most likely to exclude that LRP1^-/-^ MEFs exhibit a general defect in the endocytosis machinery.

### Binding of TcdA to LRP1

**Figure 4:**
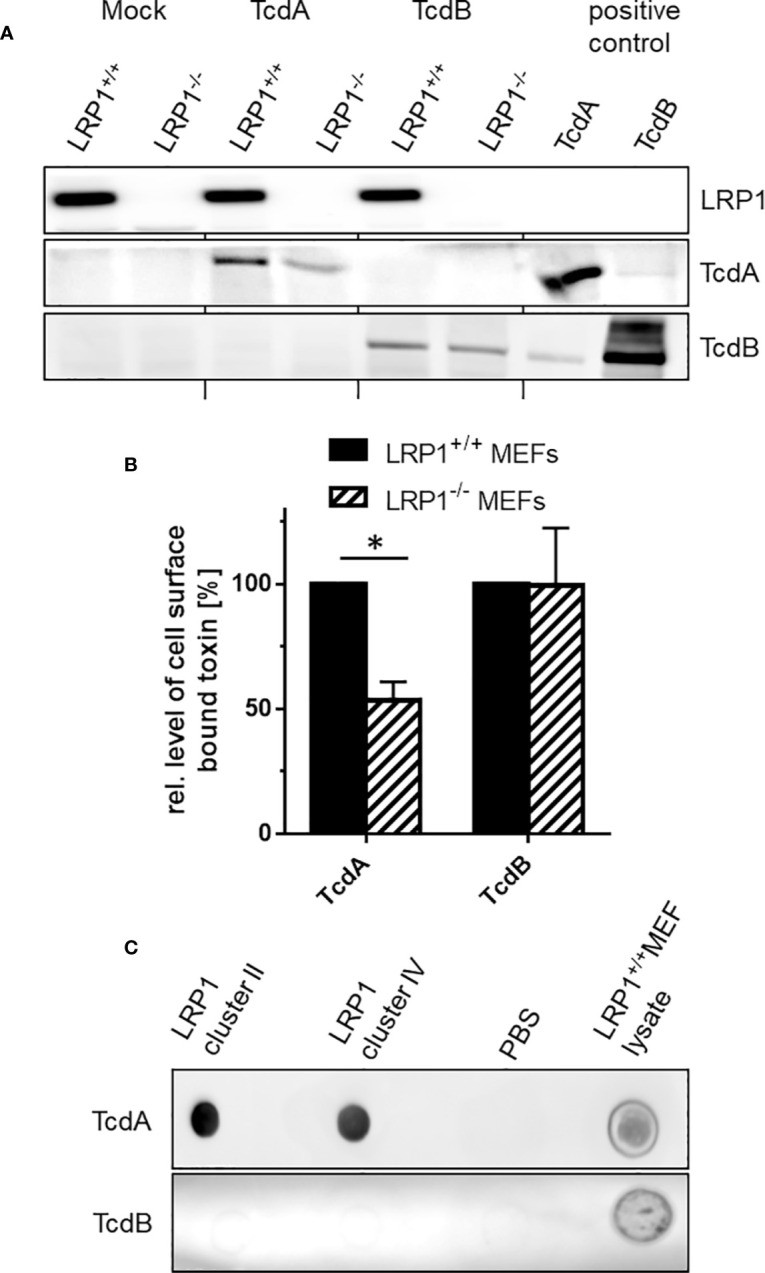
Binding of TcdA and TcdB to the cell surface. **(A)** Binding of TcdA and TcdB to LRP1^-/-^ mouse embryonic fibroblasts (MEFs) and LRP1^+/+^ MEFs was allowed at 4°C for 1 **h**. Unbound toxin was removed by washing with buffer. Cells with bound toxins were lysed and cell surface bound toxins were detected by immunoblotting. **(B)** Bound TcdA and TcdB were quantified using LabImage 1D software. Values are the mean ± SD from three independent experiments. **(C)** LRP1 cluster II and IV and lysates from LRP1^+/+^ MEFs were immobilized on nitrocellulose and exposed to solutions of TcdA (3 nM) or TcdB (3 nM) for 1 h. Bound toxin was detected using toxin specific antitoxin antibodies. * indicates significant difference, p < 0,05 as analyzed using student´s t-test.

Cell surface binding is the initial step in the uptake mechanism of the *C. difficile* toxins (Papatheodorou et al., 2018). Next, the hypothesis is followed that TcdA binds cell surface exposed LRP1. We confirmed above that LRP1 was present on the surface of fibroblasts on the cell surface (**Figure 1A**), corroborating published data (Herz et al., 2009; Emonard et al., 2014). Cell surface binding of TcdA was analyzed in the fibroblasts with blocked endocytic processes upon a shift of temperature to 4°C. Cell surface bound toxins were detected using immunoblot analysis using polyclonal antibodies to the toxins (Genth et al., 2000). In fact, TcdA bound to the cell surface of fibroblasts, corroborating published data (Olling et al., 2011). In turn, genetic deletion of LRP1 might result in reduced cell surface binding of TcdA, which turned out to be true: TcdA still bound to LRP1^-/-^ MEFs but the amount of bound TcdA was decreased by about 50% as compared to LRP1^+/+^ MEFs (**Figures 4A, B**). Expectedly, also TcdB bound to the surface of MEFs. In contrast to TcdA, the amounts of TcdB bound to LRP1^-/-^ MEFs and LRP1^+/+^ MEFs was comparable, i.e., independent of the presence of LRP1 (**Figures 4A, B**). The presence of LRP1 in LRP1^+/+^ MEFs and the absence of LRP1 in LRP1^-/-^ MEFs was confirmed using immunoblot exploiting anti-LRP1 antibody (**Figure 4A**). These observations (i) excluded a role of LRP1 in cell surface binding of TcdB and (ii) showed that reduced cellular uptake of TcdA (**Figure 3**) coincided with reduced cell surface binding.

Many LRP1 ligands bind to LRP1 cluster II and IV (Bres and Faissner, 2019). To evaluate if TcdA binds to LRP1 cluster II and IV, TcdA binding to LRP1 cluster II (covering amino acids 786 to 1,165) and IV (covering amino acids 3332 to 3779) immobilized on nitrocellulose was detected by Western blot analysis using polyclonal antitoxin antibodies. Under these conditions TcdA bound to LRP1 cluster II and IV to a comparable extent (**Figure 4C**). In contrast, TcdB bound to neither cluster II nor cluster IV (**Figure 4C**). Binding of TcdA and TcdB to lysates of LRP1^+/+^ MEFs that expressed LRP1 (**Figure 4A**) served as a positive control (**Figure 4C**). Taken together, these observations suggest that TcdA (but not TcdB) binds to LRP1 at the cell surface.

## Discussion

LRP1 ligands include growth factors, coagulation factors, extracellular matrix proteins (including matrix metalloproteases), viral proteins and toxins (Lillis et al., 2008). We here show that TcdA bound to LRP1 cluster II and IV (**Figure 4C**). This is consistent with the notion that most LRP1 ligands bind to cluster II and IV (Bres and Faissner, 2019). Furthermore, the related TpeL has been shown to bind to cluster IV as well (Schorch et al., 2014). Unfortunately, we failed in fixing the LRP1–TcdA complex by exploiting chemical cross-linkers, as in the presence of cross-linkers resulted in rapid LRP1 degradation (**Figure S4**).

The observations of this study further show that LRP1 undergoes rapid internalization into fibroblasts and Caco-2 cells (**Figure 1**). Furthermore, genetic deletion of LRP1 results in reduced cellular uptake of TcdA, as analyzed in terms of the surrogate markers Rac/Cdc42 glucosylation and PAK de-phosphorylation (**Figure 3**) and in terms of toxin-induced actin depolymerization (**Figure 2**). In sum, our observations show that TcdA binds LRP1 and that LRP1 is internalized. LRP1 thus seems to be sufficient for facilitating TcdA uptake. These findings allow the conclusion that TcdA functions as a LRP1 ligand that exploits constitutive endocytosis of LRP1 for internalization into the cell. This view is fully consistent with the well-established function of LRP1 as an endocytic receptor that clears its extracellular ligands [including lipoproteins, coagulation FVIII, and (matrix-) metalloproteinases] and thereby contributes to the homeostasis of lipid metabolism and coagulation and to the integrity of the extracellular matrix.

Genetic deletion of LRP1 results in reduced cell surface binding of TcdA by about 50% (**Figures 4A, B**), which allow the conclusion that TcdA binds LRP1 present at the cell surface. On the other hand, the finding that TcdA still bound to the surface of LRP1^-/-^ MEFs shows that additional receptor structures of TcdA (distinct from LRP1) are available at the surface of LRP1^-/-^ MEFs. Several TcdA receptors have been proposed, including gp96, Lewis I, X and Y glycans, and sulfated glucosaminoglycans (Tucker and Wilkins, 1991; Na et al., 2008; Hennen et al., 2013; Tao et al., 2019). From the observation that TcdA (albeit at higher concentrations) is capable of entering LRP1^-/-^ MEFs, we conclude that there are other endocytic receptors that facilitates TcdA uptake in the absence of LRP1. Likely candidates for endocytic receptors are members of the LDLR family.

LRP­1 associates with other membrane associated proteins on the same cell, which allows LRP1 to modulate the activity or internalization of diverse receptors including PDGF receptor, NMDA receptor subunits, TGF­β receptors, Frizzled­1, and various integrins. LRP1 also associates with gp96 (Basu et al., 2001; Binder and Srivastava, 2004). Downregulation of gp96 or inhibition of gp96 by antibodies partially blocks the biological effects of TcdA (Na et al., 2008). We here show that gp96 was present at the surface of fibroblasts and Caco2- cells without exhibiting detectable internalization (**Figure 1A, C**). Gp96 Internalization was also not observed in the presence of TcdA (**Figure 1B, D**), likely excluding that TcdA triggers its own uptake by facilitating internalization of its receptors. Gp96 thus might serve as a non-endocytic receptor of TcdA. Given that gp96 binds TcdA, association of gp96 and LRP1 might facilitate LRP1 binding of TcdA followed by TcdA internalization mediated by LRP1-mediated endocytosis. A tiny fraction of gp96 that is taken up mediated by LRP1 might escape detection in our assay.

Recently, we and others have speculated that TcdB might exploit LRP1 as an endocytic receptor for cellular uptake (Guo et al., 2017; Schottelndreier et al., 2018). We here show that genetic deletion of LRP1 affected neither TcdB-induced actin de-polymerization (**Figure 2**) nor cellular uptake (**Figure 3**) nor cell surface binding of TcdB (**Figure 4**). These observations are most likely to exclude a role of LRP1 in the cellular uptake of TcdB. Comparable observations have earlier been reported for TcdB with partially deleted receptor binding domain (Schorch et al., 2014). Our biochemical internalization assay directly proves the abundance of the receptor inside the cell (**Figure 1**). Exploiting this assay, we earlier provided evidence that neither of the TcdB receptor candidates CSPG4, PVRL3, and Frizzled 1/2/7 exhibit internalization in the presence or absence of TcdB (Schottelndreier et al., 2018). We therefore have classified CSPG4, PVRL3, and Frizzled 1/2/7 as non-endocytic receptors of TcdB. To our best knowledge, the endocytic receptor of TcdB remains to be elucidated.

**Figure 5:**
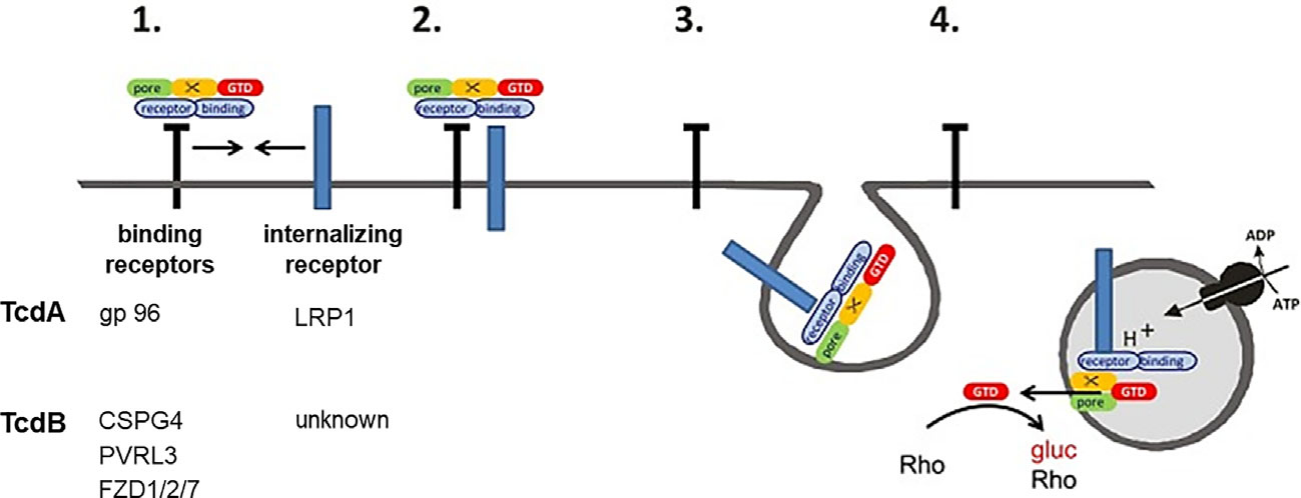
Two receptor model of TcdA and TcdB. (1) Toxin binds to (non-internalizing) binding receptors. (2) The binding receptors form a heterodimer with an internalizing receptor. (3) The toxin is released from the binding receptor, allowing internalization of the toxin internalizing receptor-complex into the early endosome. (4) The glucosyltransferase (GTD) is released into the cytosol upon acidification. The GTD mono-O-glucosylates Rho-GTPases and thereby inactivates them. Gp96, glycoprotein 96; LRP1, low density lipoproten receptor related protein 1; CSPG4, chondroitin sulfate proteoglycane; PVRL3, poliovirus receptor-like3; FZD1/2/7, Frizzled protein 1/2/7.

This study strongly supports the recently proposed two receptor model of the large clostridial glucosylating toxins (Schottelndreier et al., 2018). This model states that the toxins exploit both non-endocytic and endocytic protein receptors for cellular uptake (**Figure 5**): Cellular uptake is initiated by (presumably low affinity) binding of the toxin to the non-endocytic receptor, allowing toxin enrichment of at the cell surface. The non-endocytic receptor associates with the endocytic receptor, facilitating toxin binding to the endocytic receptor and toxin internalization by endocytosis mediated by the endocytic receptor. In sum, the observations of this study suggest that LRP1 serves as an endocytic receptor for TcdA. gp96 might rather serve as a non-endocytic receptor for TcdA.

## Data Availability Statement

All datasets presented in this study are included in the article/**Supplementary Material**.

## Author Contributions

HG and RL conceived the study. DS and AL performed the experiments. HG, RL, and DS analyzed the data and wrote the manuscript. All authors contributed to the article and approved the submitted version.

## Funding

This work was funded by the Federal State of Lower Saxony, Niedersächsisches Vorab (VWZN3215/ZN3266).

## Conflict of Interest

The authors declare that the research was conducted in the absence of any commercial or financial relationships that could be construed as a potential conflict of interest.

## Supplementary Material

Supplementary Figure 1 Internalization of LRP1 in murine fibroblasts. Internalization of reversibly biotinylated cell surface proteins (transferrin receptor (TfR); glycoprotein 96 (gp96); LDL related protein 1 (LRP1)) into murine fibroblasts was induced by temperature shift to 37°C. Cells were either left untreated (total) or exposed to glutathione to remove cell surface biotin (internalized). Biotinylated proteins were retrieved using neutravidin agarose and analyzed by immunoblotting. Representative immunoblots are shown and quantified using Labimage 1D software.

Supplementary Figure 2 Delayed TcdA-induced actin de-polymerization upon genetic deletion of LRP1. **(A)** LRP1^-/-^ MEFs and LRP1^+/+^ MEFs were treated with the indicated concentrations of TcdA **(A)** and TcdB **(B)** for 4h. Toxin-induced actin de**-**polymerization was quantified in terms of the number of rounded per total cells. Values are the mean ± SD from three independent experiments. * indicates significant differences, p<0,05 as analyzed using student´s t-test.

Supplementary Figure 3 Delayed TcdA-induced Rac/Cdc42 glucosylation upon genetic deletion of LRP1. LRP1^-/-^ MEFs and LRP1^+/+^ MEFs were treated with the indicated concentrations of TcdA (A) or TcdB (B) for 4h and relative amounts of non-glucosylated Rac/Cdc42 versus total Rac1 was quantified using Labimage 1D software and expressed as the mean ± SD from three independent experiments. * indicates significant differences, p<0,05 as analyzed using student´s t-test.

Supplementary Figure 4 Crosslinking of LRP1 in MEFs. Subconfluent MEFs were treated with 5 mM of the chemical crosslinker disuccinimidyl suberate (DSS) for 45 min at room temperature. The cells were lysed and subjected to immunoblotting.
