# Supplementary figures and images for: Low Density Lipoprotein Receptor-Related Protein-1 (LRP1) Is Involved in the Uptake of Clostridioides difficile Toxin A and Serves as an Internalizing Receptor
Source: Front Cell Infect Microbiol. 2020 Oct 19;10:565465. doi: 10.3389/fcimb.2020.565465 (PMC7604483; doi:10.3389/fcimb.2020.565465)

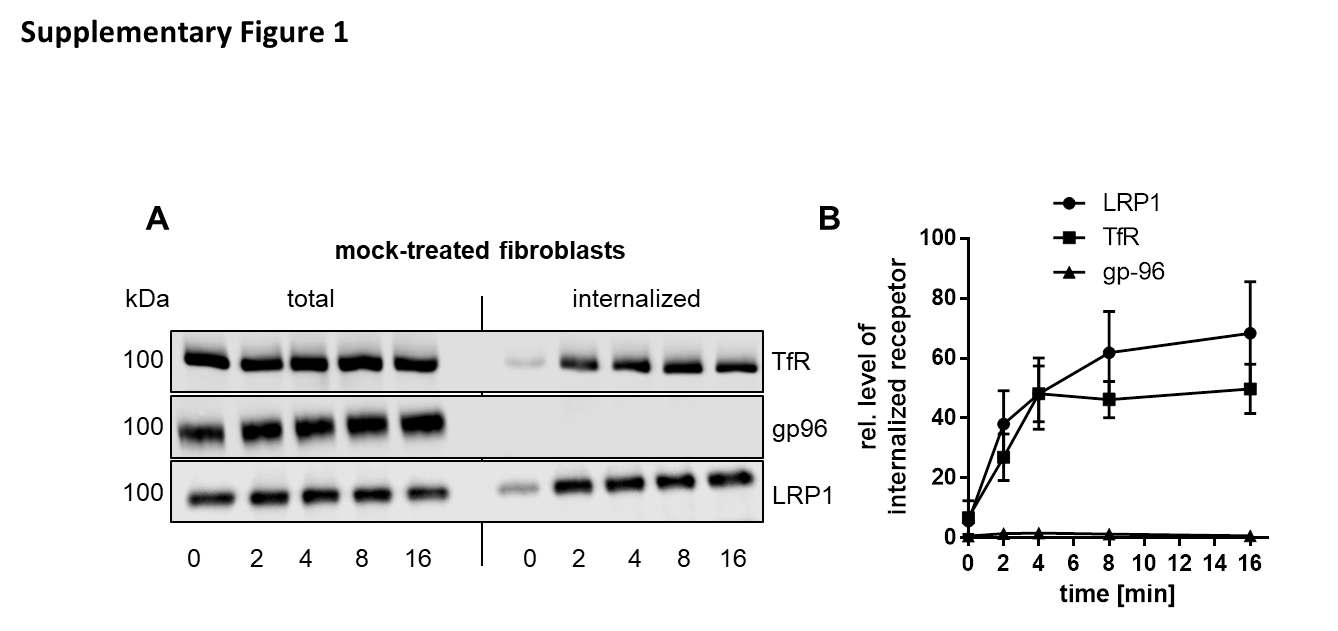

Supplement: Supplementary Figure 1 — Internalization of LRP1 in murine fibroblasts. Internalization of reversibly biotinylated cell surface proteins (transferrin receptor (TfR); glycoprotein 96 (gp96); LDL related protein 1 (LRP1)) into murine fibroblasts was induced by temperature shift to 37°C. Cells were either left untreated (total) or exposed to glutathione to remove cell surface biotin (internalized). Biotinylated proteins were retrieved using neutravidin agarose and analyzed by immunoblotting. Representative immunoblots are shown and quantified using Labimage 1D software. [file Image_1.tif]

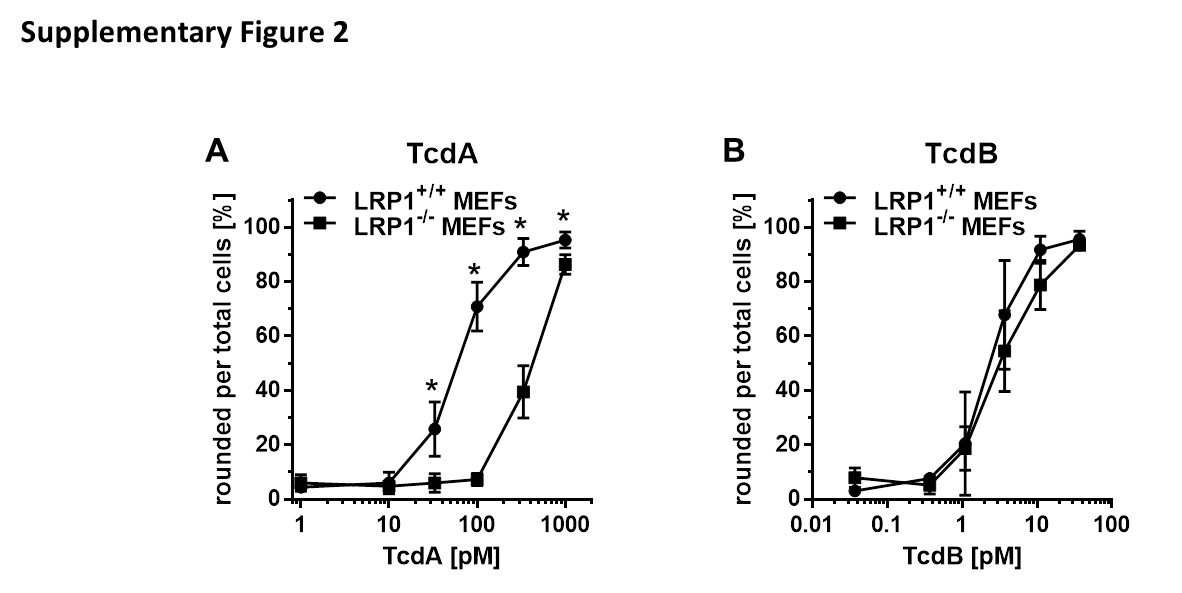

Supplement: Supplementary Figure 2 — Delayed TcdA-induced actin de-polymerization upon genetic deletion of LRP1. (A) LRP1-/- MEFs and LRP1+/+ MEFs were treated with the indicated concentrations of TcdA (A) and TcdB (B) for 4h. Toxin-induced actin de-polymerization was quantified in terms of the number of rounded per total cells. Values are the mean ± SD from three independent experiments. * indicates significant differences, p<0,05 as analyzed using student´s t-test. [file Image_2.tif]

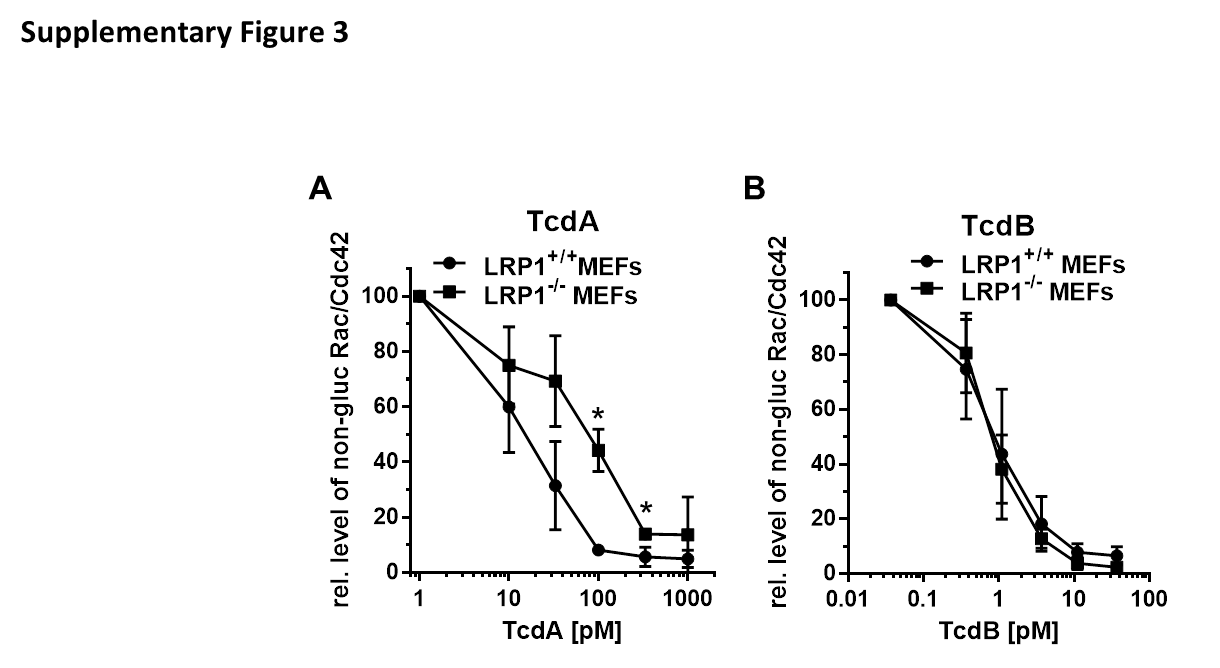

Supplement: Supplementary Figure 3 — Delayed TcdA-induced Rac/Cdc42 glucosylation upon genetic deletion of LRP1. LRP1-/- MEFs and LRP1+/+ MEFs were treated with the indicated concentrations of TcdA (A) or TcdB (B) for 4h and relative amounts of non-glucosylated Rac/Cdc42 versus total Rac1 was quantified using Labimage 1D software and expressed as the mean ± SD from three independent experiments. * indicates significant differences, p<0,05 as analyzed using student´s t-test. [file Image_3.tif]

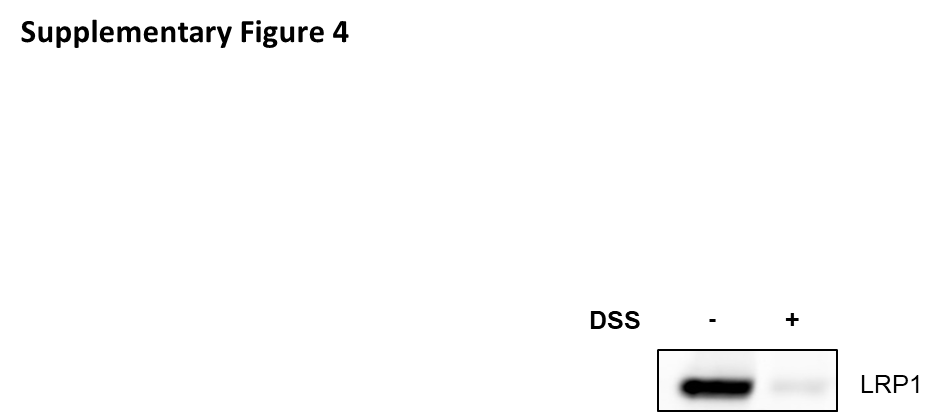

Supplement: Supplementary Figure 4 — Crosslinking of LRP1 in MEFs. Subconfluent MEFs were treated with 5 mM of the chemical crosslinker disuccinimidyl suberate (DSS) for 45 min at room temperature. The cells were lysed and subjected to immunoblotting. [file Image_4.tif]
